# Delayed presentation of a rare and complex multi-vessel pulmonary arteriovenous fistula following penetrating chest-wall trauma

**DOI:** 10.1093/jscr/rjaa605

**Published:** 2021-02-19

**Authors:** Aman B Williams, Lauren G Lax

**Affiliations:** Department of Vascular Surgery, Gold Coast University Hospital, Southport, Australia; Intensive Care Unit, Gold Coast University Hospital, Southport, Australia

## Abstract

The formation of a pulmonary arteriovenous malformation (PAVM) following penetrating chest trauma is a rare occurrence, often rapidly fatal or requiring emergency surgery. Even rarer still, is for the malformation to formed by an entangled and grossly tortuous network of multiple arteries and veins, with symptoms and eventual presentation to a medical facility taking place years after the initial injury. Without substantial literature available, and nil regarding instances of more than one involved artery and vein, we present the case of a complex left-sided PAVM, becoming symptomatic 15 years after a chest-wall stabbing. Clinical examination revealed hypoxia, tachycardia and an extracardiac bruit—prompting delineation with a computed tomography pulmonary angiogram and revealing the PAVM. Subsequent attempt at endovascular embolization was unsuccessful, though interrogation provided vital planning information for workup of urgent open-surgical ligation and resection. Surgery carries high morbidity and mortality, as does natural progression if left undealt.

## INTRODUCTION

Pulmonary arteriovenous malformation (PAVM) following penetrating chest trauma is a rare occurrence, complicated by potential life-threatening consequences. With scant appearance in current literature, it tends to occur between a single thoracic artery and vein. At time of writing, there are no reports of PAVM’s consisting of a large network of multiple feeding and draining vessels. We present this case to stimulate modern awareness, whilst highlighting the potential subtlety of this condition, it is red flags, and the multidisciplinary dilemmas in investigating and treating such a dangerous lesion.

**Figure 1:**
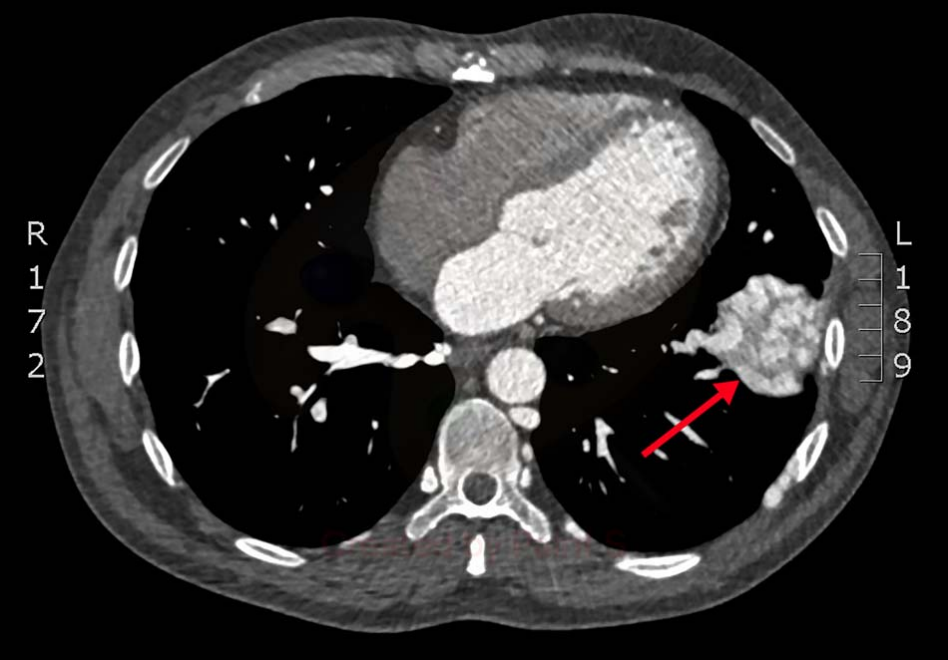
Axial slice from the CTPA, demonstrating the large, complex PAVM in the left lung’s lower lobe (red arrow).

**Figure 2:**
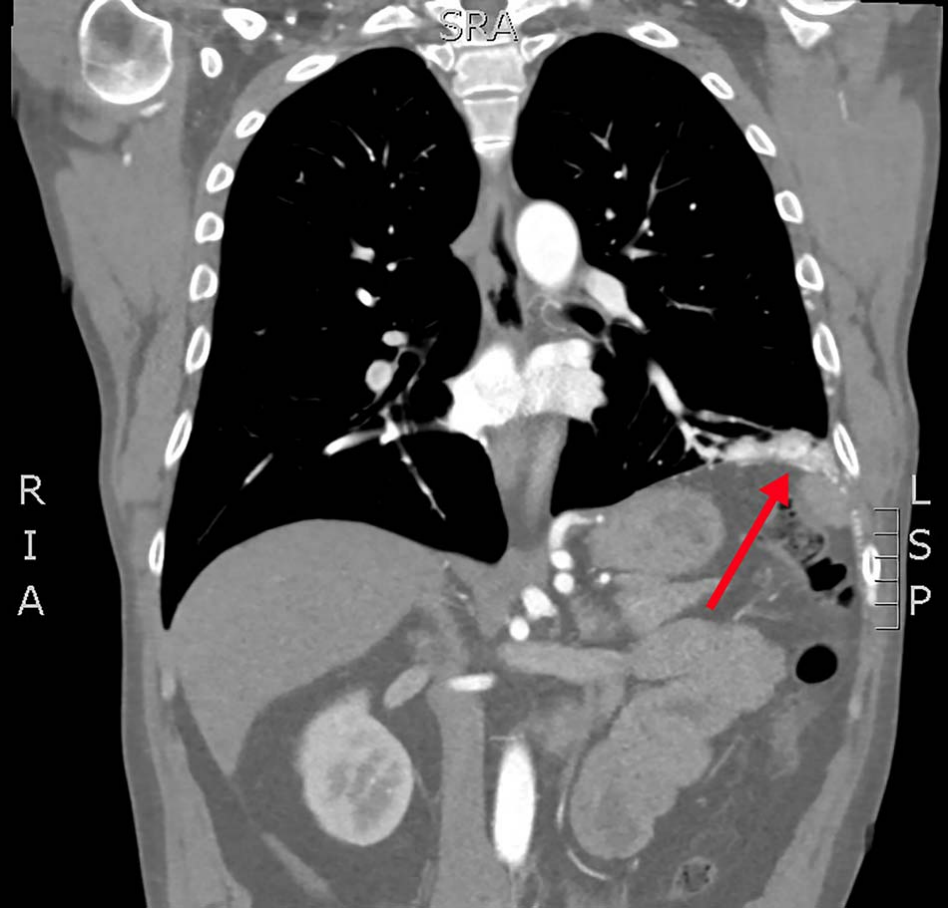
Coronal slice from the CTPA, demonstrating the large, complex PAVM in the left lung’s lower lobe, with some subdiaphragmatic extension (red arrow).

## CASE REPORT

A 42-year-old gentleman presented to hospital with chronic left chest pain, and a newfound shortness of breath on exertion (SOBOE). With no regular medications, his only past medical history to report was having been stabbed multiple times in his left anterior chest 16 years ago, undergoing wound care management at a remote hospital—complicated by a superficial infection and a prolonged course of antibiotics. On examination he appeared fit, though borderline tachycardic and oxygenating at 95% on room-air. His air entry was equal and symmetrical, however notably he had a pansystolic murmur aberrantly located below the level of the nipple—corresponding to his stab wound scar keloid. The remainder of his physical examination was unremarkable, and a plain erect chest-X-Ray yielded no further information.

**Figure 3:**
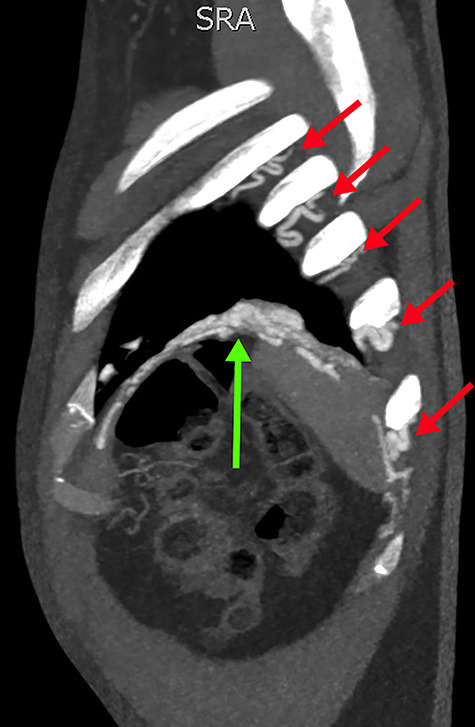
Sagittal slice from the CTPA, demonstrating tortuous intercostal arteries (red arrows) heading towards and feeding the PAVM in the left lung’s lower lobe (green arrow).

Concerned for pulmonary embolism (PE), a computed tomography pulmonary angiogram (CTPA) was arranged. PE was excluded; however, it did reveal a complex left-sided intra- and extrathoracic vascular malformation within the anteromedial segment of the left lung’s lower lobe, the lingula segment, the subdiaphragmatic space and the left chest-wall (Figs. 1 and 2). The arterial supply derived from large tortuous vessels arising from a combination of the coeliac trunk, six posterior intercostal arteries and the left inferior phrenic artery (Fig. 3). It then communicated with the left upper and lower lobe subsegmental pulmonary arteries, and the left lower lobe pulmonary vein. Subsequent echocardiography was normal, with no significant valvular pathology—evidencing that the pansystolic murmur was an arteriovenous bruit.

**Figure 4:**
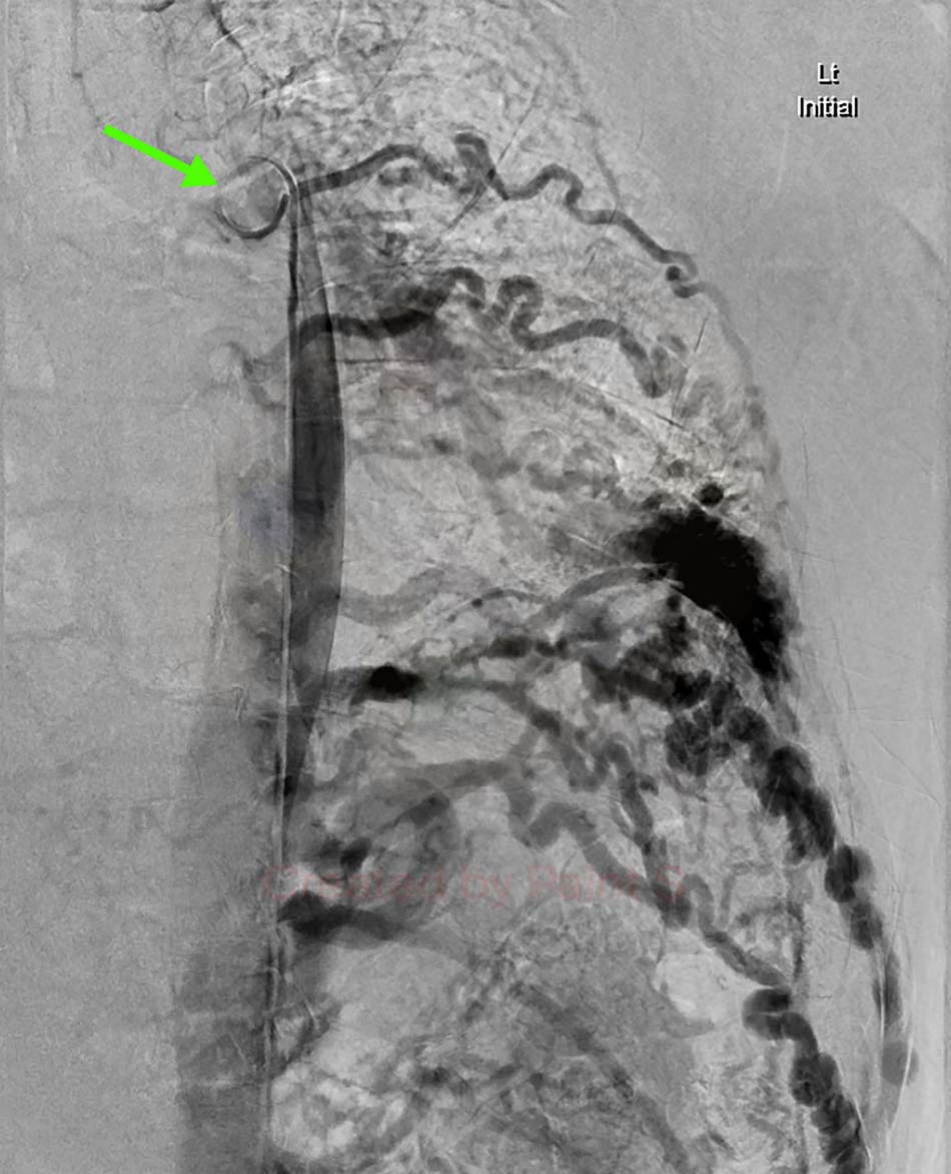
Aortic DSA using a pigtail catheter (green arrow), demonstrating extensive arterial collateralization between the left chest-wall intercostal arteries and the left lower lobe PAVM.

**Figure 5:**
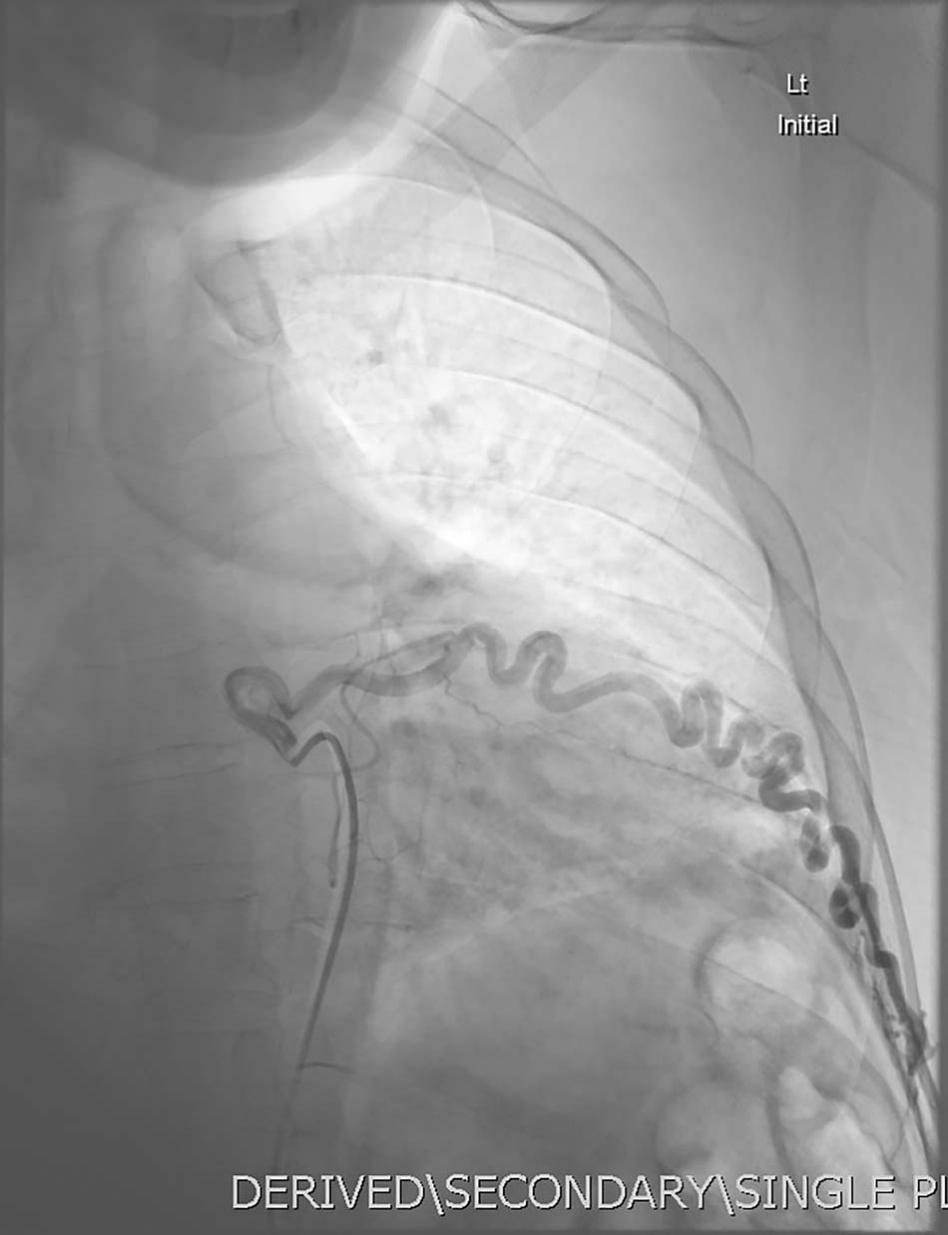
DSA utilizing superselective catheterization to demonstrate contribution to the PAVM via one of the many tortuous intercostal arteries.

**Figure 6:**
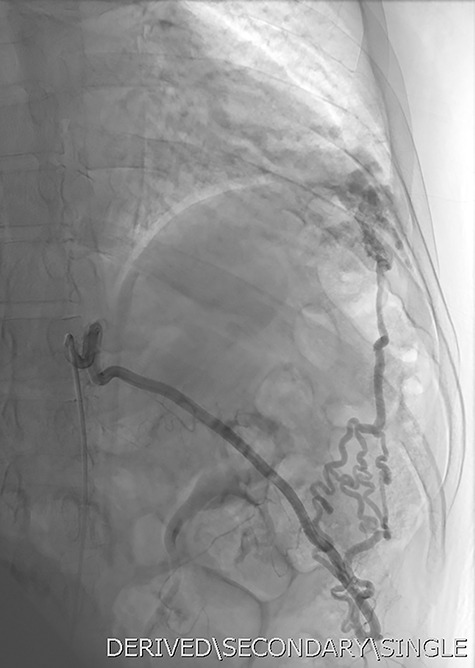
DSA utilizing superselective catheterization to demonstrate contribution to the PAVM via the left inferior phrenic artery, and its extensive subdiaphragmatic collateralization.

Given the findings, he was referred to Interventional Radiology for a formal digital subtraction angiogram with view of transcatheter embolization. Under local anaesthetic and sedation, a 5-french sheath was placed in the left common femoral artery. Using a 5-french pigtail catheter, aortic angiography was undertaken, confirming the CTPA findings and demonstrating predominant supply of the vascular malformation via the left intercostal arteries and the left inferior phrenic artery (Fig. 4)—draining into the left inferior pulmonary artery and vein. Superselective catheterization of the left intercostal arteries (Fig. 5) and the left inferior phrenic artery (Fig. 6) are shown. Unfortunately, due to the malformation’s volume and complexity, embolization was abandoned. He was subsequently referred to Cardiothoracic Surgery, currently awaiting consideration and workup for ligation and resection via video-assisted thoracoscopy, and potentially thoracotomy.

## DISCUSSION

Penetrating thoracic trauma resulting in a PAVM is a rare phenomenon, with sparse representation in modern literature. It is likely that the lack of evidence stems from pulmonary vasculature trauma being rapidly fatal, with emergency surgery carrying significant morbidity and mortality [1]. Another reason for the rarity of post-traumatic PAVM’s is due to the lower pressure gradient between the pulmonary arteries and pulmonary veins (versus systemic circulation), therefore reducing the likelihood of arteriovenous fistulation [2, 5]. Other causes of acquired PAVM’s include actinomycosis, pulmonary schistosomiasis, hepatic cirrhosis and metastatic carcinoma [2]. Though this gentleman did not describe current acute infective symptoms, previous complicating infection at time of original injury could have impaired wound and vessel remodelling, predisposing fistula formation.

Penetrating chest trauma and new respiratory decompensation are key to the history, furthermore any clinical findings of cyanosis, dyspnoea, clubbing and reported haemoptysis should raise suspicion [3]. Our patient’s chronic chest pain and SOBOE certainly are vague, though a PE must not be overlooked when these symptoms are coupled with inexplicable hypoxemia and tachycardia. The auscultated extracardiac bruit too is rare given the above-mentioned low-pressure gradient between the pulmonary arteries and veins. It is hypothesized that with the gross arterial collateralization from the engorged intercostal arteries and inferior phrenic artery, this perhaps was enough to increase this pressure gradient, hence accentuating an audible bruit.

Although unyielding in our patient, chest X-ray (in the limited available literature) occasionally indicated disease, with the presence of a round density, or other intra-pulmonary opacification (e.g. shrapnel) nearby the PAVM [3]. Needing CTPA to ultimately detect pathology, a single artery and single vein usually are the culprit link—a stark contrast to the complex system arteries and veins providing inflow and outflow to the PAVM found in our patient. Computed tomography proved incredibly useful in workup of this patient, particularly in surgical planning—not only to prompt initial endovascular interrogation (versus open surgery) but plan the access vessel approach (i.e. neck versus groin, arterial versus venous). Adjunctive to CTPA, echocardiogram served several purposes in this case. Specifically, it excluded valvular pathology, but also facilitated assessment for evidence of high-output heart failure associated with massive high-flow arteriovenous fistulae—as occasionally seen in the haemodialysis patient cohort [4].

Though treatment with minimally invasive transcatheter embolization would have been ideal, given the large number of involved vessels, endovascular salvage poses significant risks. In the rare instance that a PAVM undergoes embolization (even in patients with fewer involved vessels), return of symptoms secondary to vessel recanalization is not unheard of [5]. With multiple vessels involved, the rate of recanalization is assumed higher, where repeat intervention would compound the overall exposed iatrogenic risk to the patient. Cardiothoracic repair with thoracoscopic ligation, open thoracotomy-ligation or lobectomy have too been reported with varying success [6]. Though both endovascular and open repair may confer a measurable improvement in the patient’s degree of hypoxaemia, the risks remain significant and potentially catastrophic—including haemo-pneumothorax, cardiac tamponade, pneumonia, massive haemoptysis and death. To date, there is no evidence to suggest that open or endovascular approaches offer better outcomes in the setting of a multi-vessel PAVM’s, though adopting an ‘endovascular first’ approach provides detailed diagnostic information, and opportune (and potentially therapeutic) embolization.
